# Unusual Indirect Transmission of Pasteurella multocida Causing Bacteremia in an Immunocompetent Patient

**DOI:** 10.7759/cureus.114176

**Published:** 2026-08-08

**Authors:** Sravani Kamatam, Swathi Mogulla, Bindu Bathina, Ashish K Roy, Moni Roy

**Affiliations:** 1 Internal Medicine, Order of St. Francis (OSF) Saint Francis Medical Center, Peoria, USA; 2 Internal Medicine, St. Dominic Hospital, Jackson, USA; 3 Internal Medicine, University of Illinois College of Medicine Peoria, Peoria, USA

**Keywords:** bacteremia, gram negative bacteremia, immunocompetent, non-bite transmission, pasteurella

## Abstract

*Pasteurella multocida* is a gram-negative coccobacillus commonly found in the oral and respiratory flora of cats and dogs. Human infection typically occurs following animal bites or scratches and most commonly presents as skin and soft tissue infection. Respiratory infections and bacteremia are less common and are usually reported in elderly or immunocompromised individuals. We present a case of a previously healthy male with a one-week history of fever, nonproductive cough, fatigue, myalgias, chills, and generalized weakness. Workup revealed left lower lobe pneumonia and Pasteurella bacteremia. Further history revealed ownership of a household cat and routine cleaning of the cat’s feeding dishes with the same cloth used for personal dishes, representing a possible route of indirect exposure. The patient was treated with intravenous piperacillin-tazobactam followed by oral trimethoprim-sulfamethoxazole, with subsequent clearance of bacteremia and complete radiographic resolution of pneumonia. Pasteurella infections are most associated with animal bites and scratches; however, non-bite transmission through animal saliva and contaminated fomites has been reported. Pneumonia with bacteremia is an uncommon manifestation, particularly in young immunocompetent hosts. This case highlights an unusual presentation of *Pasteurella multocida* bacteremic pneumonia in a healthy young adult and underscores the importance of obtaining a detailed animal exposure history, including indirect exposures, when evaluating patients with invasive Pasteurella infections.

## Introduction

Pasteurella species are small, gram-negative coccobacilli that are common commensals and pathogens of domestic and wild animals. Human infection most frequently occurs following cat or dog bites, scratches, licks, or exposure to animal nasopharyngeal secretions, while indirect transmission or exposure through wildlife is rare. *Pasteurella multocida* is the species most commonly implicated in human disease. Severe infections are typically associated with immunocompromised states and underlying comorbidities, including cirrhosis, diabetes mellitus, chronic kidney disease, and malignancy [1].

Clinical manifestations of Pasteurella infection most commonly include skin and soft tissue infections, followed by bone and joint infections. Less frequently, the organism can cause respiratory tract infections, bacteremia, meningitis, endocarditis, and other invasive infections. Pasteurella pneumonia is predominantly reported in older adults with chronic pulmonary disease, alcoholism, cirrhosis, malignancy, or other immunocompromising conditions [2]. In contrast, bacteremic pneumonia occurring in a young, immunocompetent individual without traditional risk factors is exceedingly rare and has been described only in a limited number of cases in the literature.

## Case presentation

A 21-year-old male with no significant past medical history presented to the Emergency Department with a one-week history of fever, nonproductive cough, generalized weakness, fatigue, myalgias, palpitations, and chills. He denied tobacco use, significant alcohol consumption, illicit drug use, and had no known sick contacts. His past surgical history was notable only for an appendectomy.

On presentation, the patient was febrile with a maximum temperature of 103.2°F and tachycardic with heart rates ranging from 110 to 120 beats per minute and a respiratory rate of 28, and oxygen saturation was 99% on room air with no need for any supplemental oxygen. Physical examination revealed a well-appearing male in no acute distress with dry mucous membranes. Cardiovascular exam showed regular rhythm with tachycardia, and lung auscultation revealed bilateral rhonchi. No skin rashes or lesions were observed.

Initial laboratory evaluation demonstrated leukocytosis of 14.23 × 10³/µL with neutrophilic predominance (absolute neutrophil count 12.38 × 10³/µL). Comprehensive metabolic panel was notable for mild hyperglycemia (glucose 125 mg/dL) and a slight elevation in total bilirubin (1.3 mg/dL) and an isolated low IgM level of 20 mg/dl as shown in Table 1. The respiratory pathogen assay test for common viral and bacterial organisms, included in Table 2, group A streptococcus testing and mononucleosis screening were negative. Chest radiography revealed focal airspace consolidation in the posterior segment of the left lower lobe.

**Table 1:** Admission Laboratory Investigations and Reference Ranges

Laboratory Test	Admission Value	Reference Range
WBC (×10³/µL)	14.23	4.0–11.0
Absolute Neutrophil Count (×10³/µL)	12.38	1.5–7.5
Glucose (mg/dL)	125	70–140 (random)
Total Bilirubin (mg/dL)	1.3	0.2–1.2
IgM (mg/dL)	20	40–230

**Table 2:** Respiratory Pathogen Array

Pathogen Category	Pathogen Tested	Result
Adenovirus	Adenovirus	Not Detected
Seasonal Coronaviruses	Coronavirus 229E	Not Detected
Seasonal Coronaviruses	Coronavirus HKU1	Not Detected
Seasonal Coronaviruses	Coronavirus NL63	Not Detected
Seasonal Coronaviruses	Coronavirus OC43	Not Detected
SARS-CoV-2	SARS-CoV-2	Not Detected
Human Metapneumovirus	Human Metapneumovirus	Not Detected
Rhinovirus/Enterovirus	Rhino/Enterovirus	Not Detected
Influenza Viruses	Influenza A	Not Detected
Influenza Viruses	Influenza A (H1)	Not Detected
Influenza Viruses	Influenza A (H3)	Not Detected
Influenza Viruses	Influenza A (2009 H1)	Not Detected
Influenza Viruses	Influenza B	Not Detected
Parainfluenza Viruses	Parainfluenza Virus 1	Not Detected
Parainfluenza Viruses	Parainfluenza Virus 2	Not Detected
Parainfluenza Viruses	Parainfluenza Virus 3	Not Detected
Parainfluenza Viruses	Parainfluenza Virus 4	Not Detected
Respiratory Syncytial Virus	Respiratory Syncytial Virus (RSV)	Not Detected
Bacterial Respiratory Pathogens	Bordetella pertussis	Not Detected
Bacterial Respiratory Pathogens	Bordetella parapertussis (IS1001)	Not Detected
Bacterial Respiratory Pathogens	Chlamydia pneumoniae	Not Detected
Bacterial Respiratory Pathogens	Mycoplasma pneumoniae	Not Detected

**Figure 1:**
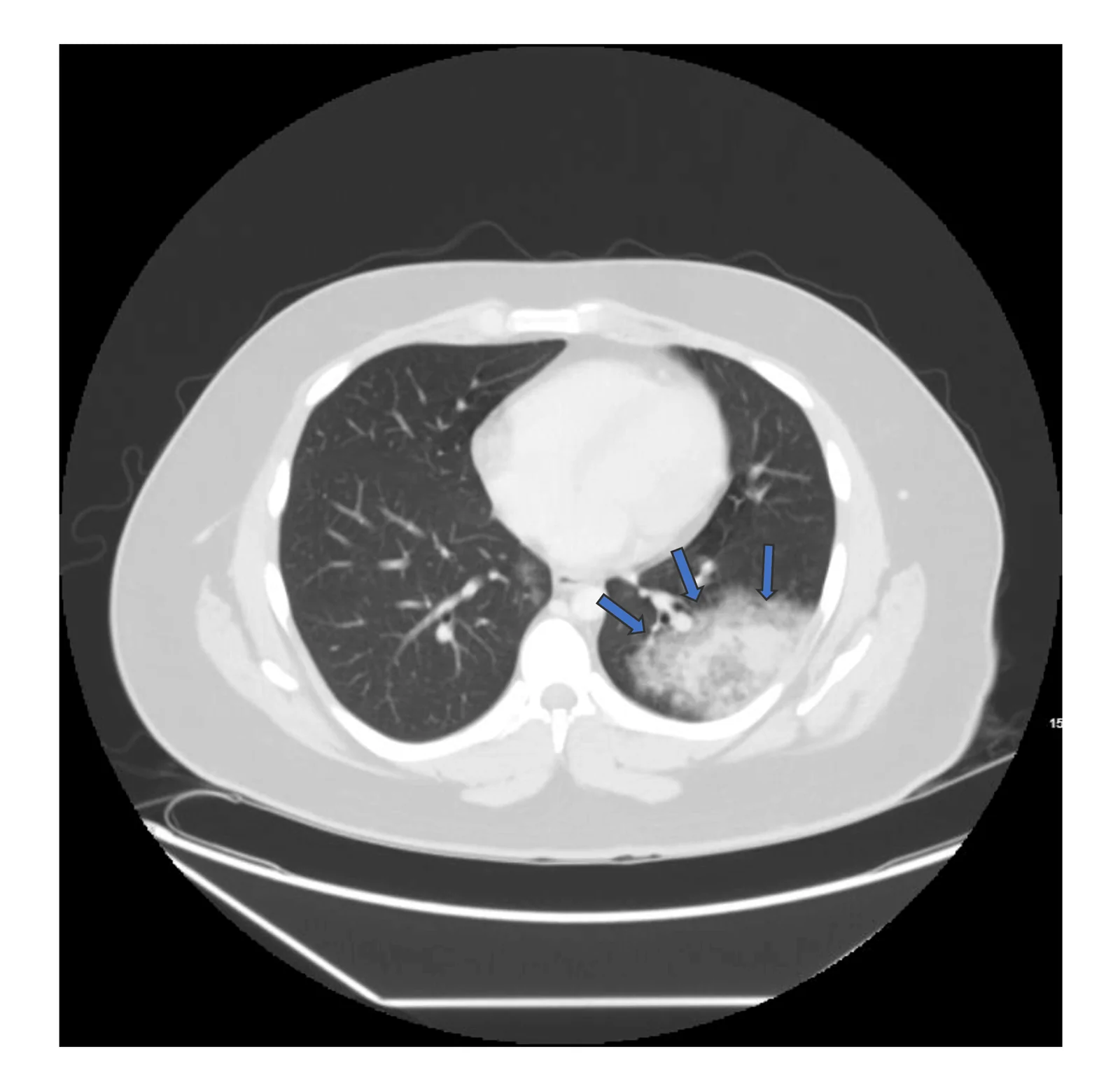
CT Scan of Chest, Abdomen and Pelvis with Contrast Demonstrating Left Lower Lobe Airspace Consolidation

The patient was initially started on amoxicillin for presumed community-acquired pneumonia as an outpatient. Upon hospital admission, antibiotic therapy was escalated to piperacillin-tazobactam. Blood cultures obtained on admission (2/2 sets) grew *Pasteurella multocida*, sensitive to ampicillin, tetracycline, and trimethoprim-sulfamethoxazole, as shown in Table 3. Computed tomography of the chest, abdomen, and pelvis confirmed left lower lobe pneumonia, as shown in Figure 1, without evidence of an alternative source of bacteremia. HIV antigen/antibody testing was negative. Immunoglobulin levels were measured, with IgA at 174 mg/dL, IgM at 20 mg/dL, and IgG at 636 mg/dL. Although a low IgM level (20 mg/dL) was identified during the acute illness, repeat testing demonstrated improvement, suggesting that this was a transient abnormality rather than evidence of a persistent humoral immunodeficiency.

**Table 3:** Susceptibility Testing for Pasteurella multocida

Testing Method	Antimicrobial Agent	Zone of Inhibition (mm)	Interpretation
Kirby–Bauer Disk Diffusion	Ampicillin	27	Susceptible
Kirby–Bauer Disk Diffusion	Linezolid	22	Interpretation Not Available
Kirby–Bauer Disk Diffusion	Tetracycline	25	Susceptible
Kirby–Bauer Disk Diffusion	Trimethoprim/Sulfamethoxazole	26	Susceptible

The patient's close contact with a household cat and use of a shared dishcloth for cleaning both pet and personal dishes represented a plausible source of indirect exposure. Infectious Diseases was consulted and confirmed that the bacteremia was likely secondary to this exposure.

The patient was treated with intravenous piperacillin-tazobactam with subsequent clearance of blood cultures. He was discharged on oral trimethoprim-sulfamethoxazole to complete a total of 10 days of antimicrobial therapy, based on susceptibility testing.

At outpatient follow-up, repeat immunologic testing demonstrated improvement in IgM levels. A follow-up chest radiograph obtained one month after hospitalization showed complete resolution of the left lower lobe consolidation. 

## Discussion

Pasteurella species are gram-negative coccobacilli commonly found in the upper respiratory tract of animals, with carriage rates of approximately 70-90% in cats and 20-50% in dogs [3]. These organisms cause a variety of diseases in animals, including pneumonia, hemorrhagic septicemia, fowl cholera, and localized infections.

Human infection most commonly occurs following dog bites, cat scratches, or bites. However, non-bite transmission has also been described, including exposure through animal licks, kissing animals, or sharing food. Additional mechanisms such as wound licking, contamination of plantar ulcers by animal saliva, and transmission via contaminated fomites have also been reported [4]. Other animals, including horses, pigs, rats, rabbits, and wolves, may serve as sources of infection.

In humans, Pasteurella infections can present with a wide spectrum of clinical manifestations, including cellulitis, pneumonia, septic arthritis, meningitis, peritonitis, bacteremia, and endocarditis. A review of 79 patients suggested that non-bite transmission is more common in elderly individuals, and that severe, life-threatening infections are more likely in patients with underlying comorbidities [3]. Bite- and scratch-related infections typically produce an intense inflammatory response with cellulitis and purulence, and in rare cases may progress to necrotizing fasciitis [5].

Septic arthritis caused by *Pasteurella multocida *typically involves joints proximal to the site of inoculation, most commonly the knee, and shows a predilection for previously damaged joints such as those affected by rheumatoid arthritis, degenerative joint disease, or prosthetic joints [6].

Although pneumonia is the most common respiratory manifestation, Pasteurella can also cause other respiratory tract infections, including pharyngitis, sinusitis, otitis media, mastoiditis, empyema, and lung abscess. Severe pulmonary infections are more frequently observed in elderly, immunocompromised patients or those with underlying chronic lung disease [7].

Pasteurella meningitis has been reported primarily in infants and adults older than 60 years. Proposed mechanisms include direct inoculation through bites or licking of scalp abrasions, as well as hematogenous spread [8].

In the present case, infection is suspected to have occurred through indirect exposure via a washcloth contaminated by a cat. Diagnosis is established by isolation of the organism in culture. Gram stain and aerobic and anaerobic cultures should be obtained from wounds, and respiratory specimens should be collected when pulmonary involvement is suspected.

Treatment options for Pasteurella infections include penicillins, fluoroquinolones, third-generation cephalosporins, carbapenems, tetracyclines, and trimethoprim-sulfamethoxazole, as Pasteurella species are generally susceptible to these agents and are typically resistant to vancomycin and clindamycin. Because Pasteurella infections most commonly occur following animal bites or scratches, empiric therapy is usually selected to provide coverage for both Pasteurella and other organisms commonly found in dog and cat bite wounds. Recommended regimens include beta-lactam/beta-lactamase inhibitor combinations, such as ampicillin-sulbactam, amoxicillin-clavulanate, or piperacillin-tazobactam. Alternative regimens include a fluoroquinolone, doxycycline, or trimethoprim-sulfamethoxazole combined with anaerobic coverage, typically metronidazole or clindamycin.

Localized infections are typically treated for 7-10 days, whereas more severe infections require 10-14 days. Surgical interventions such as abscess drainage, debridement, or joint washout may be necessary, followed by prolonged antibiotic therapy of 4-6 weeks in cases such as septic arthritis [9].

Prognosis depends on the site and severity of infection. While most soft tissue infections resolve with appropriate therapy, osteomyelitis may result in delayed healing or residual deformity. Mortality rates of 25-30% have been reported in severe infections such as meningitis, bacteremia, and endocarditis [7].

This case is unique in demonstrating an uncommon mode of indirect transmission through a contaminated personal hygiene item in an immunocompetent individual, highlighting the potential for non-bite exposures to result in clinically significant infection and contributing to the growing body of literature on atypical routes of Pasteurella transmission.

## Conclusions

*Pasteurella multocida* infection should be considered in patients with animal exposure even in the absence of a bite or scratch, as indirect transmission through contaminated fomites or household items may occur. Pasteurella pneumonia with bacteremia can develop in immunocompetent individuals without significant comorbidities, although it is more commonly reported in elderly or immunocompromised patients. A thorough exposure history, including pet ownership and nontraditional animal contact, is essential in identifying potential sources of Pasteurella infection and guiding appropriate antimicrobial therapy.
